# Central nervous system involvement in chronic lymphocytic leukemia: a case report and review of literature

**DOI:** 10.1186/s13256-024-04639-4

**Published:** 2024-07-10

**Authors:** Li-Yuan Qin, Ye Geng, Jian-Feng Mu, Wen-Jun Wang, Cai-Xia Zhang, Yi-Nan Gao, Jian-Xia He

**Affiliations:** 1 Department of Hematology, Shanxi Provincial People’s Hospital, Taiyuan, 030012 China; 2 Department of Medicine, Shanxi Vocational College of Tourism, Taiyuan, 030091 China

**Keywords:** Chronic lymphocytic leukemia, Central nervous system, Ibrutinib, Pomalidomide

## Abstract

**Background:**

Central nervous system involvement in chronic lymphocytic leukemia rarely occurs, and there is no standard therapy for central nervous system involvement in chronic lymphocytic leukemia. This article aims to analyze the diagnosis and treatment of central nervous system involvement in chronic lymphocytic leukemia.

**Case presentation:**

It reports two cases of central nervous system involvement in chronic lymphocytic leukemia describing the clinical course, therapy, and prognosis. Case 1 is a 67-year-old Asian male patient, he experienced complications with central nervous system involvement after developing resistance to ibrutinib, bendamustine, and rituximab (BR) chemotherapies. The central nervous system lesion was controlled with high-dose methotrexate combined with pomalidomide, but Richter transformation occurred several months later. Case 2 is a 62-year-old Asian female patient, she had central nervous system involvement at initial diagnosis, and bone marrow and central nervous system lesions were controlled by ibrutinib therapy.

**Conclusion:**

Central nervous system involvement in chronic lymphocytic leukemia is rare and can be diagnosed on the basis of clinical features, cerebrospinal fluid testing, and radiographic evaluation. Ibrutinib, pomalidomide, and other drugs that can cross the blood–brain barrier may be effective for treating central nervous system involvement in chronic lymphocytic leukemia.

## Introduction

Chronic lymphocytic leukemia (CLL) is a type of blood and bone marrow cancer that primarily affects older adults, with 70% of CLL patients being over 65 years old [1]. CLL is characterized by the proliferation and accumulation of small mature-appearing lymphocytes in the blood, bone marrow, and lymphoid tissues, representing a monoclonal B lymphocyte proliferative disease. However, involvement of the central nervous system (CNS) in CLL, known as CLL-CNS, is a rare occurrence. Neurological complications resulting from CNS involvement have been reported in only 1% of patients with CLL. On the basis of a review of relevant literature, this article reports two cases of patients with CLL-CNS. After a comprehensive diagnosis based on clinical symptoms and medical examinations, we explored the use of drugs that can cross the blood–brain barrier, such as ibrutinib and pomalidomide, to control the disease.

## Case presentation

### Case 1

A 67-year-old Asian male patient was previously admitted to the hematology department in January 2018 owing to a persistent elevation in leukocyte count for more than 3 years. Peripheral blood examination revealed a lymphocyte count of 37.15 × 10^9^/L and platelet count of 109 × 10^9^/L. Bone marrow morphology showed obvious hyperactive hyperplastic lymphocyte proliferation (64%). Further examination of the bone marrow biopsy revealed active hyperplasia and extensive distribution of lymphocytes. Flow cytometry immunotyping indicated that 55.44% of the B lymphocytes exhibited abnormal B lymphocytes, expressing CD5, immunoglobulin D (IgD), IgM, and Ig-Kappa, partially expressing CD200, and weakly expressing CD20, CD19, CD81, CD22, and CD79b, but not expressing CD11c, FMC7, CD103, CD10, CD23, CD38, CD25, CD138 and CD43. These findings were consistent with the phenotypic characteristics of CD5+CD10− cell lymphoma. Additional testing using fluorescence in situ hybridization (FISH) demonstrated *TP53* gene deletion and next-generation sequencing revealed a *TP53* gene mutation and unmutated *IGHV* gene. The patient had a karyotype of 46,XY [12]. On the basis of these findings, the patient was diagnosed with chronic lymphocytic leukemia. Since there was no indication for treatment at the time, the patient was followed up every 2–6 months.

In October 2018, during a blood re-evaluation, the patient’s lymphocyte count was found to be 76.51 × 10^9^/L. However, by December 2018, the count had further increased to 106 × 10^9^/L, leading to the patient’s admission and initiation of treatment with ibrutinib at a daily dose of 420 mg. In September 2019, ibrutinib was discontinued owing to gastrointestinal bleeding. Following the patient’s recovery from surgery, ibrutinib was restarted, and the patient achieved complete remission (CR) as evaluated by bone marrow flow cytometry and routine blood examination. However, in March 2021, the disease showed signs of progression again, as evidenced by a steady increase in leukocyte count, and significant splenomegaly observed during physical examination. At the end of April 2021, the lymphocyte count reached 142.43 × 10^9^/L, and the platelet count was 102 × 10^9^/L. Bone marrow morphology and biopsy revealed a significant increase in lymphocytes. Flow cytometry immunotyping showed that 69.08% of B lymphocytes exhibited abnormal characteristics, expressing CD19, Ig-Kappa, CD20, IgD, IgM, CD81, and CD5, partially expressing CD23 and CD200, but not expressing CD103, CD10, CD38, CD25, CD43, CD11c, FMC7, and Ig-Lambda. Next-generation sequencing revealed a *TP53* gene missense mutation (heterozygous), *KRAS* missense mutation (1.3%), and a mutation ratio of less than 2% for the *IGHV* gene. Karyotyping showed chromosomes of 47,XY, add (2) (p21), + 4, − 7, 8, 13, add (19) (p13), + 3mar [6]/46,XY [14]. FISH analysis indicated *TP53* and *DLEU* gene deletions. Venetoclax was recommended, but owing to economic reasons, immunochemotherapy was considered. The patient was administered a BR regimen consisting of rituximab (600 mg on day 0) and bendamustine (150 mg on days 1–2) for four cycles, resulting in the achievement of complete remission (CR).

On 24 November 2021, the patient exhibited a sluggish response, lethargy, poor speech, and unsteadiness while walking. On 30 November the patient was admitted to the emergency department in a shallow coma. During physical examination, it was observed that the patient remained in a shallow coma and had a slow light reflection. The pain limb showed a visible retraction in response to the pain stimulus, whereas the right limb did not respond. Pathological signs were present on the right side. The sensory and motor reflexes were uncooperative, the neck was stiff, and signs of meningeal irritation were evident. The patient’s initial admission diagnosis was chronic lymphocytic leukemia with Rai stage III and Binet stage A. Additionally, the patient had a CLL-International Prognostic Index (IPI) score of 8, which indicates a very high-risk group. The presence of a *TP53* mutation, along with an unmutated* IGHV* gene and CNS involvement were considered in the diagnosis.

**Fig. 1:**
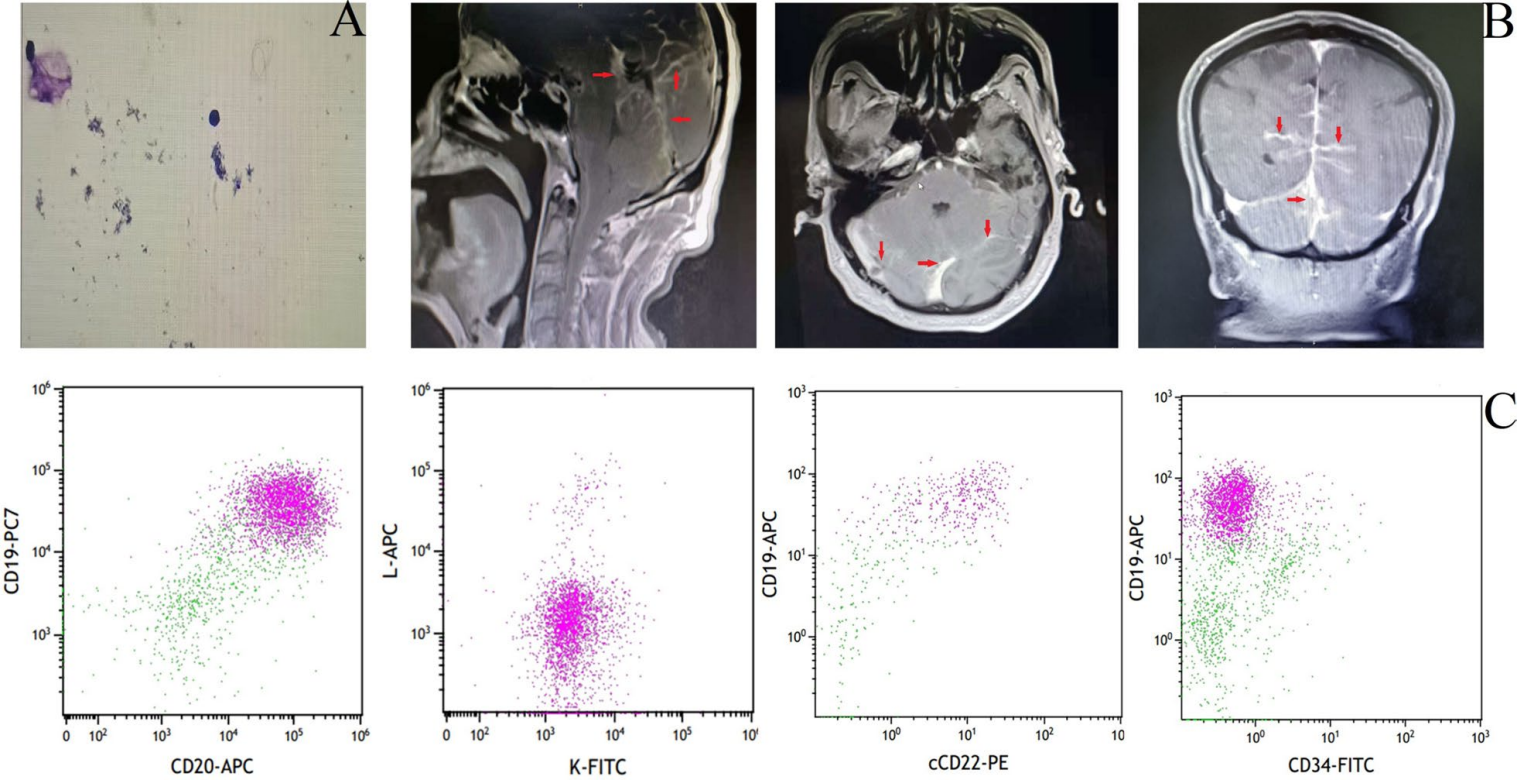
Diagnosis and central nervous system involvement in case 1. **A** Denotes the cerebrospinal fluid morphology of case 1. **B** Depicts the magnetic resonance imaging pictures. The red arrows indicate the abnormal enhancement points in the bilateral temporal occipital lobe, cerebellar hemisphere, brainstem, and basilar cisterna soft membrane. **C** Depicts the scatter plots from cerebrospinal fluid- flow cytometry immunotyping

On 30 November 2021, the patient received intrathecal chemotherapy drugs through a lumbar puncture, leading to an improvement in consciousness. The following day, on 1 December 2021, the patient was administered methotrexate at a dosage of 3 g on day 0, dexamethasone at 10 mg from day 1 to 4, and pomalidomide at 4 mg for a 21-day cycle within a 28-day period. As a result, the patient’s consciousness, drowsiness, and physical activity significantly improved. On 16 December 2021, the second course of treatment was administered, which included methotrexate at 3 g on day 0, dexamethasone at 10 mg on days 1 to 4, and pomalidomide at 4 mg for a 21-day cycle with a 28-day period. Following this, the patient regained consciousness, spoke fluently, and was subsequently discharged from the hospital. The patient continued taking oral pomalidomide at a dosage of 4 mg. Subsequently, the patient underwent three more cycles of the above treatment regimen. After two cycles, partial remission (PR) was achieved, and after four cycles, complete remission (CR) was achieved. On 19 May 2022, case 1 presented with back pain and weakness in both lower limbs that had been persistent for one week. A craniocerebral magnetic resonance imaging (MRI) scan showed abnormally enhanced signals near the posterior horn of the right ventricle. Additionally, an MRI of the lumbar spine MRI showed a new lesion in the spinal canal between the thoracic 11 and sacral 2 vertebrae. Considering the progression of the disease, after consultation with the patient’s family, palliative care was chosen as the treatment approach. The patient was administered high-dose methotrexate at a dosage of 5.5 g on day 0 and dexamethasone at 10 mg from days 1 to 4. In addition, temozolomide at a dosage of 0.1 g was given from day 1 to 5. As a result of this treatment, the patient experienced an improvement in waist and lower limb weakness and was discharged. On 20 June 2022, the peripheral blood routine examination showed a leukocyte count of 27.98 × 10^9^/L (compared with 5.91 × 10^9^/L on 30 May 2022), with 56.4% of lymphocytes showing abnormality in the bone marrow. The lymphocyte appeared large with some showing nucleolar residue. A small number of large cells with scattered distribution was noticed in the bone marrow biopsy. Immunohistochemistry indicated the following results: CD5−, CD20+, PAX5−, a small fraction of CD10+, Sox11−, C-MYC−, cyclinD1−, CD22−, partial BCL-2+, and BCL-6−. Bone marrow immunotyping revealed two groups of abnormal B lymphocytes, accounting for 48.47% of the cells. One group represented 25.69% of the cells, and the other group represented 22.78% of the cells. Both types expressed CD20, CD19, CD81, CD22, and IgM. One group expressed Ig-Lambda, while the other group expressed Ig-Kappa. CD25, CD11c, CD5, CD43, CD200, CD34, CD23, FMC7, and CD138 were not expressed. FISH analysis revealed *TP53 *gene deletion. A craniocerebral MRI scan showed a slightly enlarged lesion near the posterior horn of the right ventricle, and the lesion was observed in the left basal ganglia. A MRI of the lumbar spine showed an abnormal enhancement in the lumbar 1 cone, also a new lesion. Intrathecal administration of chemotherapy drugs was performed via a lumbar puncture (Fig. 1B). Morphological examination of cerebrospinal fluid (CSF) revealed cells with large cell bodies (Fig. 1A) and immature morphology expressing CD19, CD20, CD81, IgM, Ig-Kappa, and CD22, while not expressing CD103, CD43, CD11c, CD200, CD23, CD25, CD34, CD5, and Ig-Lambda (Fig. 1C), indicating Richter transformation. Unfortunately, the family decided to discontinue treatment at this point.

### Case 2

**Fig. 2:**
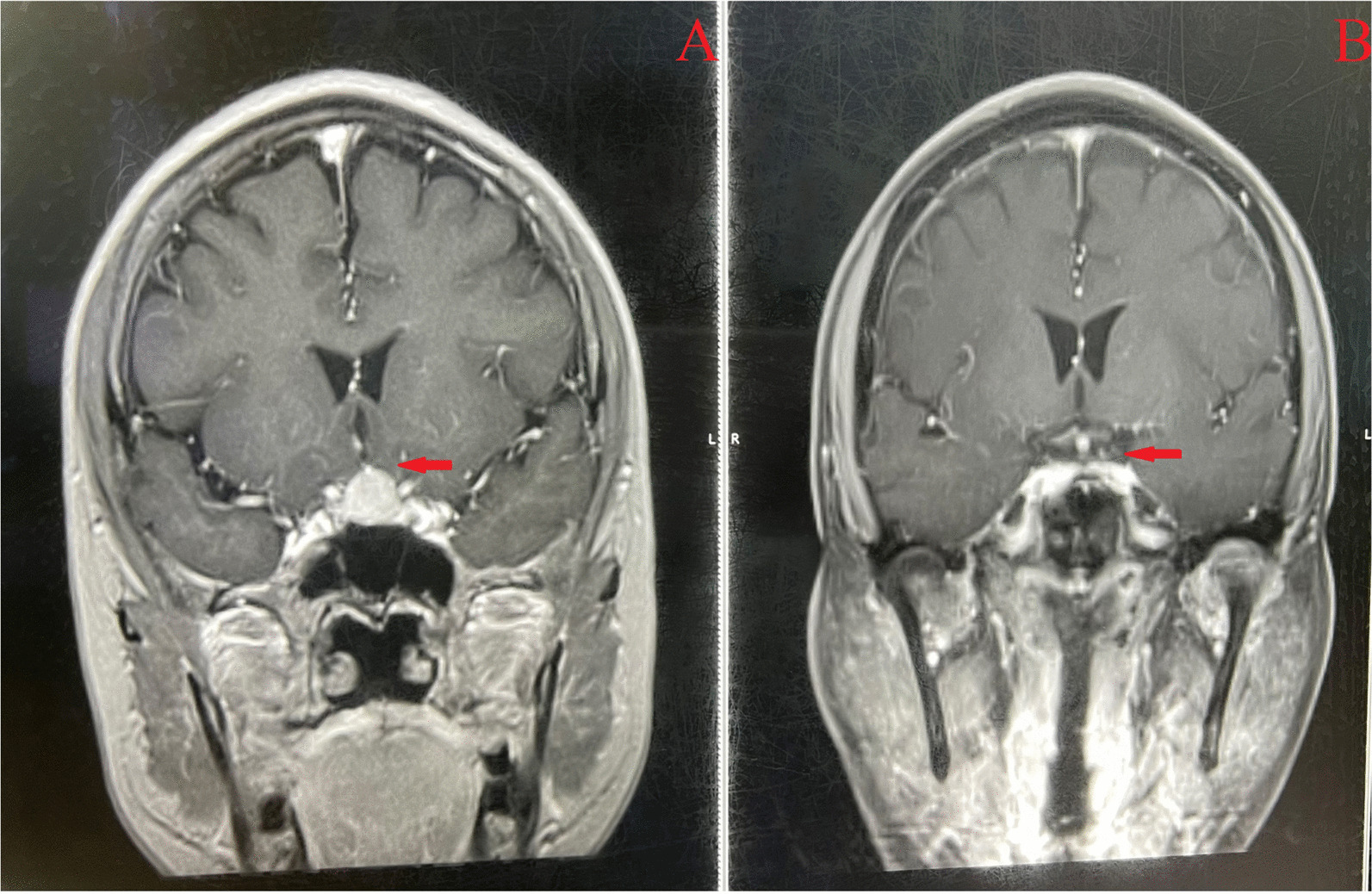
Images from the craniocerebral magnetic resonance imaging scan of case 2. **A** Shows the craniocerebral magnetic resonance imaging scan of case 2. **B** Shows the craniocerebral enhanced magnetic resonance imaging picture of case 2 after treatment. The red arrow indicates the lesion site in the stellar region

In December 2019, a 62-year-old Asian female patient experienced sudden syncope accompanied by confusion for 3–5 minutes. Upon examination at the local hospital, an elevation in leukocyte levels was observed. A craniocerebral MRI conducted the following day revealed a mass measuring approximately 2.0 cm × 3.0 cm in the stellar region (Fig. 2A). The patient also developed numbness in the extremities, swelling of both hands and left lower limb, and experienced difficulty walking and squatting. Subsequent bone marrow morphology analysis indicated an increased lymphocyte count. Owing to the persistence of intermittent dizziness for over half a year, the patient was transferred to our hospital in March 2020 for further evaluation and management.

Examination finding: The leukocyte count was 18.44 × 10^9^/L and the platelet count was 190 × 10^9^/L. Peripheral blood examination also showed an elevated proportion of mature lymphocytes (69%). Bone marrow morphology revealed hyperactive hyperplastic cells with an increased proportion of lymphocytes (69.25%), predominantly consisting of mature lymphocytes. On bone marrow biopsy, a class of cells with Pat-like or scattered distribution was observed. These cells had small cell bodies, large cell nuclei with rough chromatin, a quasi-circular shape, no nucleolus, and little cytoplasm. Immunohistochemistry analysis showed that the cells were negative for CD3, while a few cells expressed CD5+, CD20 (focal or scattered), PAX5 (focal or scattered), and Sox 11 (few focal or scattered). The cells were negative for AnnexinA1, CyclinD1, CCND1, and P53. Bone marrow immunophenotyping revealed abnormal B lymphocytes accounted for 61.27% of the population, expressing CD19, IgM, IgD, and Ig-Lambda, partially expressing CD81, CD200 and CD23, but not expressing CD103, CD10, Ig-Kappa, CD20, and CD138. Next-generation sequencing did not detect any gene mutations. FISH analysis showed negative results for *IGH/CCND3-*,* IGH* gene mutation, *IGH/CCND1*, *BCL6* gene mutation, *MALT*, *C-MYC*, and *IGH/BCL2*. Lymph node biopsy (left cervical lymph node) exhibited loss of lymph node structure and diffuse proliferation of lymphocytes, indicative of B-small cell lymphoma/CLL. Immunohistochemistry indicated the following results: CD20+, CD21+, CD23+, CD43++, LEF1+, Bcl-2+, a small fraction of CD3, CD10, Bcl-6, Mum-1, CD5, Cyclin D1, C-myc, partial P53+, Ki-67 (40%+), and SOX11−.

Diagnosis: CLL (Rai stage I, Binet stage B, and IPI score of 1) with CNS involvement.

Treatment: The patient was started on ibrutinib at a dose of 560 mg on 29 March 2020. Following treatment, the patient experienced improvements in muscle strength and tension of the limbs, resolution of edema in both lower limbs, normal gait, presence of physiological reflexes, and absence of pathological reflexes. A craniocerebral enhanced MRI scan showed a reduction in the size of the mass in the stellar region compared with the previous image (Fig. 2B). Currently, case 2 remains on ibrutinib at 560 mg without any reported discomfort.

## Discussion

CLL is known for its heterogeneous clinical manifestations [2]. While most patients experience an indolent disease course with slow progression and long survival, CLL is often associated with various immune abnormalities. These abnormalities in cellular and humoral immunity can give rise to specific complications in patients with CLL. As the disease progresses, monoclonal lymphocytes may infiltrate the lymph nodes, spleen, and liver, and occasionally other organs, such as skin [3], kidney [4], and gastrointestinal tract [5]. However, central nervous system (CNS) involvement in CLL is considered extremely rare. In a cohort study involving 4174 patients with CLL, the incidence of CNS involvement was found to be only 0.4% [6].

No systematic reviews currently exist for risk factors of CNS involvement in patients with CLL [7]. However, CNS involvement has not been correlated with the CLL disease stage, course, sex, age, immunophenotype, and high-risk chromosomal abnormalities [2, 8]. Although patients with CLL with complex chromosomal abnormalities and *TP53* gene mutations are considered to have the worst prognosis, no studies have found associations between these factors and CNS involvement in patients with CLL.

The diagnosis of CLL-CNS was based on clinical symptoms, imaging features, and CSF analysis [9, 10]. Owing to the heterogeneity of the disease, patients with CLL can present with a variety of CNS symptoms, including movement disorders, confusion, optic neuropathy, cerebellar dysfunction, cranial nerve palsy, headache, and fever. However, some autopsy studies have showed CNS involvement in patients who never exhibited CNS symptoms [11], confirming that 8–71% of patients with CLL had CNS involvement upon autopsy [12]. Clinical studies have found that approximately 1% of patients with CLL develop neurological complications owing to CNS involvement.

The imaging features of CNS involvement include diffuse coverage of the leptomeninges by leukemic cells diffusely, nodular growth, plaque-like deposition, and intraparenchymal infiltration [13–15]. However, craniocerebral MRI imaging had low sensitivity in the diagnosis of intracranial CLL and the imaging characteristics can be confused with those of meningioma [11]. In terms of the CSF analysis, CSF should be obtained through lumbar puncture, and a combination of CSF biochemistry, cell morphology, and flow cytometric detection is necessary for diagnosis. CSF cytology is considered the gold standard, with a sensitivity of 50–60% [16]. However, CSF may be contaminated by peripheral blood and CLL-B cells may be detected, resulting in misdiagnosis [6]. In 2021, a study utilized digital polymerase chain reaction (PCR) to detect circulating free tumor DNA in CSF, aiming to improve the sensitivity of tumor cell detection in CSF [17]. This method requires primers and probes for the tumor target genes and is limited by the high cost and limited routine detection.

The prognosis of CLL-CNS is related to the disease status at the time of diagnosis. A retrospective study involving 30 patients with CLL-CNS found that the 5-year overall and progression-free survivals were 72% and 43%, respectively, if CNS involvement occurred at the initial diagnosis of the disease. The progression-free survival of case 2 was more than 2 years with regular ibrutinib treatment from the initial diagnosis. However, if CNS involvement occurred during the progression of the disease, the 5-year overall survival and progression-free survival were 48% and 0%, respectively. In case 1, CNS involvement occurred during the progression of CLL and the disease progressed rapidly within 5 years [15]. In conclusion, once CNS involvement occurs in patients with CLL, less than half of the patients develop progressive CLL, leading to reduced overall survival.

Currently, the treatment for CLL-CNS varies greatly [15]. Over the past three decades, the treatment of CLL has evolved from an immunochemotherapy regimen, such as rituximab combined with fludarabine, and cyclophosphamide chemotherapy regimen (FCR) to targeted therapy, including Bruton tyrosine kinase (BTK) inhibitor, class I phosphatidylinositol 3-kinases (PI3K) inhibitor, and BCL-2 (proteins in B cell 2) inhibitors, which have greatly improved the overall survival of patients with CLL. The combination of FCR or BR (bendamustine plus rituximab chemotherapy) with intra-CSF chemotherapy (IT) has been suggested as an effective treatment for CLL.

The survival and proliferation of CLL cells strictly depend on the tumor microenvironment, which consists of macrophages, T cells, and stromal follicular dendritic cells [18, 19]. These components produce proteins, including cytokines and chemokines necessary for CLL cells’ growth and proliferation. Bruton tyrosine kinase (BTK) is a key protein in the B cell receptor signaling pathway, involved in B cell proliferation, transport, chemotaxis, and adhesion. Ibrutinib, as a BTK inhibitor, can inhibit the growth of tumor cells and promote apoptosis by regulating the tumor microenvironment, reducing the secretion of chemokines and proinflammatory factors, and downregulating the expression of anti-apoptotic proteins bcl-2 family. Furthermore, ibrutinib irreversibly inhibited BTK and induced the differentiation of CD4+ T cells into Th1 cells. Importantly, ibrutinib can cross the blood–brain barrier and is used in the treatment of lymphomas involving CNS [20]. In a retrospective study of four patients treated with ibrutinib [21], three patients cleared abnormal lymphocytes in CSF after 3 months of treatment, achieved clinical complete remission, and had no recurrence after a median follow-up of 9 months. Case 2 was associated with CNS involvement at the initial diagnosis of CLL and following regular ibrutinib treatment, the condition was stable, which was consistent with the above-mentioned study. Additionally, ibrutinib has demonstrated efficacy in the treatment of relapsed and refractory primary CNS lymphoma [22] and has been approved by the Food and Drug Administration (FDA) for first-line clinical use in relapsed and refractory primary CNS lymphoma.

Pomalidomide, as a new third-generation immunomodulator, has the ability to cross the blood–brain barrier, with a crossing rate of approximately 40% in mice. Animal studies have shown that pomalidomide is associated with significantly longer survival. It exhibits dual antilymphoma activity acting both through cytotoxicity on tumor cells by regulating the tumor immune microenvironment, specifically by polarizing the tumor-associated macrophages from the M2 type to M1 type [23]. Studies found that pomalidomide combined with dexamethasone had a significant effect on relapsed and refractory primary CNS lymphoma [23].

According to previous treatment guidelines for CLL, the first-line treatment typically involved immunochemotherapy, such as FCR and BR regimens. However, in the case of case 1, ibrutinib was selected as the first-line treatment owing to the presence of a *TP53* gene mutation. In the event of developing resistance to ibrutinib, alternative options, such as BCL-2/CD20 McAb/PI3K inhibitor, combination therapy, or chimeric antigen receptor T cell therapy (CAR-T), could be considered. Owing to low accessibility and economic reasons, we selected the BR chemotherapy regimen. After four courses of chemotherapy, the condition of case 1 became stable. However, when the patient experienced recurrent CNS symptoms, we chose pomalidomide as it effectively crosses the blood–brain barrier, unlike BCL-2/CD20 McAb. After pomalidomide treatment, the patien’s condition was stable for more than 5 months. The exploratory use of pomalidomide can be considered in patients with CLL-CNS.

Approximately 2–10% of CLL undergo Richter transformation during the course of the disease [24], resulting in the transformation into diffuse large B-cell lymphoma, Hodgkin’s lymphoma, and plasmablastic lymphoma [6]. This transformation is associated with a short survival time and poor prognosis [25]. The risk factors for Richter transformation in patients with CLL were not identified. However, factors, such as *TP53* gene mutation, *C-MYC *malformation, unmutated *IGHV* gene, and *CD38* gene polymorphism, may contribute to Richter transformation in patients with CLL [26]. It is yet to be determined whether CLL-CNS involvement is a risk factor for Richter transformation. In the case of case 1, Richter transformation occurred 5 months after CNS involvement, indicating that patients with CLL should remain vigilant for the possibility of Richter transformation following CNS involvement.

## Conclusion

CNS involvement in patients with CLL is rare. However, early recognition and treatment for CLL-CNS are crucial to improve survival and quality of life.

## Data Availability

All data generated or analyzed during this study are included in this published article.
